# Evaluation of biological and psychometric variables in children and adolescents with intellectual disabilities engaged in aquatic and land-based physical activities

**DOI:** 10.1007/s00421-026-06195-9

**Published:** 2026-05-29

**Authors:** Glykeria Kyriakidou, Anatoli Petridou, Paraskevi Giagazoglou, Vassilis Mougios, George Tsalis

**Affiliations:** 1 School of Physical Education and Sport Science at Serres, Aristotle University of Thessaloniki, 54124 Thessaloniki, Greece; 2 Laboratory of Evaluation of Human Biological Performance, School of Physical Education and Sport Science at Thessaloniki, Aristotle University of Thessaloniki, 54124 Thessaloniki, Greece

**Keywords:** Cognitive impairment, Mental retardation, Heart rate, Maturation, Salivary cortisol, α-amylase

## Abstract

**Purpose:**

The aim of the study was to examine the physiological and psychological responses to aquatic and land-based activities in children and adolescents with intellectual disability (ID) and/or autism spectrum disorder (ASD).

**Methods:**

A total of 30 participants (21 male, 9 female), aged 11.8—18.6 years and diagnosed with ID and/or ASD, completed both a swimming session and a dryland activity session. Anthropometric data, biological maturation, and heart rate (HR) responses were recorded, including the lowest (HRlow), highest (HRhigh), and mean (HRmean) values during activity. Salivary cortisol and α-amylase concentrations were measured before and after each session. Participants’ enjoyment and preference for physical activity were assessed using the Children’s Assessment of Participation and Enjoyment and Preferences for Activities of Children questionnaires.

**Results:**

HR responses did not differ significantly between activities. Cortisol decreased significantly following activity (from 0.105 ± 0.076 to 0.082 ± 0.076 µg/dL, *p* = 0.012), while α-amylase showed a non-significant decrease (from 157 ± 101 U/mL to 137 ± 112 U/mL, *p* = 0.094). Participants reported greater enjoyment of swimming than land-based activity (4.2 ± 1.0 vs. 2.5 ± 1.9, *p* < 0.001) and expressed a stronger preference for swimming (2.9 ± 0.4 vs. 2.4 ± 0.7, *p* < 0.001).

**Conclusion:**

Both swimming and land-based activities may serve as moderate-intensity activities that support reductions in physiological and psychological stress in children and adolescents with ID and ASD. Swimming appeared to be more enjoyable and preferred, possibly due to its calming environment and potential to enhance self-efficacy.

## Introduction

Intellectual disability (ID) is a neurodevelopmental disorder characterized by significant limitations in intellectual functioning and adaptive behavior, affecting everyday skills (American Psychological Association –APA 2020; Schalock et al. 2010; Tassé et al. 2012). These limitations typically include difficulties in reasoning, problem solving, learning, abstract thinking, communication, social skills, and independent daily living activities, such as personal care, decision making, and social participation (Schalock et al. 2010; Tassé et al. 2012). ID often cooccurs with other conditions, most notably autism spectrum disorder (ASD; APA 2020). Individuals with ASD experience increased social stress during adolescence, as demands for social competence increase (Ladd 2006). This stress often leads to social withdrawal, loneliness, and heightened risk for anxiety and depression (Bellini 2006; White and Roberson-Nay 2009; Kuusikko et al. 2008).

Physical activity (PA) benefits individuals with ID and ASD by improving fitness, mental health, social inclusion, and learning (Menear and Neumeier 2015). However, participation is often limited by barriers such as a lack of specialized programs and the prevalence of competitive formats (Jansiewicz et al. 2006). Additionally, motivation and sensory sensitivities often limit participation and social skill development (Zhao et al. 2018; Baker et al. 2008). Wouters et al. (2019) found that only half of the children with moderate to severe ID met the World Health Organization’s activity recommendations, highlighting the need for regular PA. Individuals with ID often have low muscle strength, balance (Blomqvist et al. 2013; Hale et al. 2009), and cardiovascular fitness due to sedentary lifestyles, which increases the risk of chronic health issues (Graham and Reid 2000; ACSM 2000). Children with ASD face high risk of obesity, partly due to low PA levels and medication effects (Rimmer et al. 2007; Hellings et al. 2001; Persico et al. 2021).

Fragala-Pinkham et al. (2008) demonstrated that aquatic exercise can improve cardiorespiratory endurance, muscular strength, and motor skills in children with disabilities, including ASD, with increased time expended within the target heart rate zone associated with faster completion of motor tasks. Tailored programs have also been linked to reduced obesity and improved fitness (Ross et al. 2001; Casey et al. 2012; Pitetti et al. 2007). These findings support the importance of individualized exercise interventions for improving health in individuals with ID and ASD.

Adolescence poses increased challenges for individuals with ASD, who often exhibit increased sensitivity to sensory stimuli and resistance to change. Corbett and Simon (2014) reported elevated physiological arousal and social stress in adolescents with ASD, indicative of increased developmental reactivity. Although PA can offer benefits, it may also exacerbate stress responses, particularly under motor, environmental, or social strain. To assess physiological stress, biochemical markers, such as cortisol and α-amylase, are commonly used (Takai et al. 2004; Chatterton et al. 1996). Cortisol, a hormone regulated by the hypothalamic-pituitary-adrenal (HPA) axis, plays a key role in energy regulation, immune function, and metabolism (Levine 2000; Bornstein et al. 2008). It exhibits a circadian rhythm and increases in response to pain or vigorous exercise but may be suppressed by endorphin release (Ma et al. 2022). Salivary α-amylase, secreted mainly by the parotid gland and comprising nearly half of salivary proteins (Sahu et al. 2014), serves as a non-invasive marker of sympathetic nervous system activation, increasing significantly during acute stress (Chatterton et al. 1996). Aerobic exercise notably raises the salivary α-amylase concentration, which typically returns to baseline within 30 min; the response is amplified with high exercise intensity and cold exposure.

Relatively few studies have examined stress biomarkers in populations with disabilities. To date, seven studies have investigated exercise interventions (Abe et al. 2002; Edmonds et al. 2015; Lundqvist et al. 2022; Pan et al. 2019; Park et al. 2020; Park and Kim 2020; Tabares et al. 2012), two studies have examined cognitive interventions (Anesiadou et al. 2021; de Vaan et al. 2020), and one study has applied a combined approach (Poquérusse et al. 2018). The findings of these studies indicate that the choice of intervention modality exerts a significant influence on the physiological response to stress. Specifically, creative and non-competitive types of exercise have been shown to reduce cortisol levels (Abe et al. 2002; Lundqvist et al. 2022; whereas competitive conditions are associated with increased α-amylase (Edmonds et al. 2015). However, only Anesiadou et al. (2021) measured salivary cortisol and α-amylase simultaneously employing an academic performance test and a moral cognition task. Furthermore, the previous studies did not compare the responses of these two biomarkers in different physical activities, nor did they examine correlations between the biomarkers themselves or between the biomarkers and biological or psychological variables.

Based on the aforementioned shortcomings in the literature, the aims of the present study were (i) to compare the physiological (as addressed through heart rate, salivary cortisol, and salivary α-amylase) and psychological (through enjoyment and activity preference questionnaires) responses of children and adolescents with ID and/or ASD between aquatic and land-based activities, and (ii) to examine the relationships between physical activity type, stress biomarkers, and perceived enjoyment.

## Materials and methods

### Participants

An a priori sample size calculation was conducted using G*Power 3.1.9.7 software (Universität Düsseldorf, Germany), as described by Faul et al. (2007). Assuming a medium effect size (partial η² = 0.137; Cohen 1988) for two-way repeated-measure ANOVA with two activities (swimming and land-based) and two time points (pre- and post-activity) a sample size of 15 was deemed sufficient to detect significant differences with a statistical power of 0.95, an α level of 0.05, and a correlation coefficient of 0.5 between repeated measures. However, in view of potential dropouts—particularly due to the uncertainty regarding completion of the study by individuals with disability—we decided to recruit 30 participants.

The study involved 30 children and adolescents of both sexes, aged 11.8 to 18.6 years and diagnosed with ID or ASD, who participated in both aquatic and land-based physical activities regularly (at least once a week for each type of activity) for the past six months. All participants had more than one year of experience in swimming and dryland activity programs and were able to understand and follow specific activity instructions. Individuals with health issues or those unable to participate in vigorous physical activity were excluded.

### Ethics

The study was approved by the Bioethics and Ethics Committee of the Department of Physical Education and Sport Science at Serres, Aristotle University of Thessaloniki (approval number: ERC-016/2022) and was conducted in accordance with the principles of the Declaration of Helsinki. All participants and their legal guardians were informed both verbally and in writing about the study’s purpose, methodology, and potential risks. Informed written consent was obtained from the guardians.

### Study design

Each participant completed two 46-minute physical activity sessions: one swimming session, was conducted in a 25-meter pool with a water temperature of 27 °C and an ambient temperature of 22–25 °C, and one dryland activity session took place in an indoor gymnasium (40 × 20 m) maintained at 22–25 °C. The swimming session consisted of (i) a 10-minute warm-up (freestyle and backstroke), (ii) a 30-minute main workout that included swimming drills using a kickboard with alternating arm freestyle or backstroke, as well as dolphin or breaststroke kicks, and (iii) a 6-minute cool-down. The session totaled 500 m. The dryland session included (i) a 15-minute warm-up consisting of 800 m of brisk walking, (ii) a 25-minute main workout featuring selected station-based exercises using light weights and a Pilates ball, and (iii) a 6-minute cool-down involving stretching exercises. Although the girls’ menstrual phase was not formally monitored, the measurements were conducted during the interval between menstrual periods. The sessions were conducted in a random counterbalanced order on separate days, with at least 48 h of rest between them.

### Measurements

Anthropometric data were collected with the participants barefoot, using a Seca 213 stadiometer (Hamburg, Germany) and a Xiaomi Mi Body Composition Scale 2 (Beijing, China). Biological maturation was assessed using Tanner stage images (Marshall and Tanner 1969), which were shown to the participants’ guardians.

Given the need for adapted, population-specific approaches when assessing exercise intensity in individuals with intellectual disabilities and autism spectrum disorder, an ID-specific maximal heart rate (HRmax) formula proposed by Fernhall and Pitetti (2001) was used (HRmax = 189 − 0.59 × age). Heart rate was continuously monitored using the waterproof Polar Verity Sense sensor and the Polar H10 heart rate monitor (Polar Electro, Kempele, Finland), both connected wirelessly to the Polar Flow application. Continuous heart rate data were recorded, including the lowest (HRlow), highest (HRhigh), and mean (HRmean) heart rates. Relative HRhigh and HRmean values were expressed as percentages of estimated HRmax, which was calculated using the formula, HRmax = 189–0.59 × age (Fernhall and Pitetti 2001). Activity time at six intensity zones based on heart rate (< 50%, 50–59.9%, 60–69.9%, 70–79.9%, 80–89.9%, and 90–100% of HRmax) was also recorded.

Saliva samples for cortisol and α-amylase measurements were collected at baseline (rest) and within 5 min of the completion of the physical activity sessions. Participants were instructed not to eat, drink, chew gum, or brush their teeth for 60 min prior to saliva sampling. When this was unavoidable, they were instructed to rinse their mouth thoroughly with cold water 5 min before sample collection. The participants collected unstimulated (via passive drool technique) approximately 5 mL of saliva in plastic Falcon tubes. Then, about 1.5 mL from each saliva sample was transferred with a plastic Pasteur pipette into an Eppendorf tube and stored in a portable refrigerator until the completion of the session. Finally, the samples were stored in an ultra-freezer at − 80 °C until analysis. Salivary cortisol and α-amylase concentrations were measured using enzyme-linked immunosorbent assay kits (TECAN, IBL, Germany), following the manufacturer’s instructions. Absorbance readings were obtained using a Rayto RT-2100 C photometer (Shenzhen, China), and all kit controls were within the acceptable ranges specified in the manufacturer’s QC certificate.

Children’s participation in daily activities was assessed using standardized questionnaires focusing on physical, play, and school-related activities, which can be completed by children, parents, or professionals through interviews (Anastasiadi, 2013). Participation in leisure and social activities was evaluated using the Children’s Assessment of Participation and Enjoyment (CAPE) and the Preferences for Activities of Children (PAC), instruments widely used in children with intellectual disabilities and autism spectrum disorder (Potvin et al. 2013; Solish et al. 2010; Lami et al. 2018). Both questionnaires employ a self-report format, allowing for the assessment of children’s subjective enjoyment and activity preferences, which may differ from parental reports. The CAPE and PAC demonstrate strong predictive validity and have been successfully adapted and validated cross-culturally, including in Greek populations (King et al. 2004, 2007; Anastasiadi, 2013). Participants’ enjoyment and preference for physical activity were assessed on a day before the activities using the CAPE and PAC questionnaires, respectively. Enjoyment was rated on a five-point Likert scale, while preference was assessed using a three-point scale supported by pictorial representations of related activities. Responses were categorized into four dimensions: swimming enjoyment, land-based activity enjoyment, swimming preference, and land-based activity preference.

### Statistical analysis

Descriptive data are presented as mean and standard deviation. The distribution of all dependent variables was assessed using the Shapiro-Wilk test, and no significant deviations from normality were found. To compare activity time across intensity zones and activities, two-way repeated-measures ANOVA (activity × zone) was conducted. Cortisol and α-amylase concentrations were analyzed using two-way repeated-measure ANOVA (activity × time). When a significant interaction was found, simple main effect analysis followed, with Bonferroni correction applied for multiple comparisons. Effect sizes of ANOVA outcomes were determined as partial η^2^ and were classified as small (0.01–0.058), medium (0.059–0.137), or large (> 0.137) according to Cohen (1988). To compare HRlow, HRhigh, HRmean, activity time at six intensity zones, and questionnaire responses between the swimming and land-based activities, paired-sample Student’s *t* tests were employed. Pearson correlation analysis was used to examine associations between variables. The level of statistical significance was set at α = 0.05 for all analyses. All analyses were performed using SPSS version 27.0 (IBM SPSS Statistics, Armonk, NY).

## Results

The demographic and anthropometric characteristics of the participants are presented in Table 1.

**Table 1:** Demographic and anthropometric characteristics of the participants (mean ± standard deviation)

Disability	ID		ASD	
Sex	Male (*n* = 10)	Female (*n* = 5)	Male (*n* = 11)	Female (*n* = 4)
Age (y)	15.9 ± 2.2	15.4 ± 2.6	13.7 ± 2.3	15.1 ± 2.8
Tanner stage	3.0 ± 1.2	3.4 ± 1.8	2.1 ± 1.3	4.0 ± 0.8
Height (m)	1.61 ± 0.1	1.68 ± 0.2	1.66 ± 0.1	1.62 ± 0.1
Weight (kg)	60.8 ± 18.9	69.2 ± 21.7	71.3 ± 24.5	56.1 ± 7.4
BMI (kg/m^2^)	23.2 ± 5.1	24.1 ± 4.8	25.2 ± 6.1	21.2 ± 1.6

*ASD:* autism spectrum disorder; *ΒΜΙ:* body mass index; *ID:* intellectual disability

**Fig. 1:**
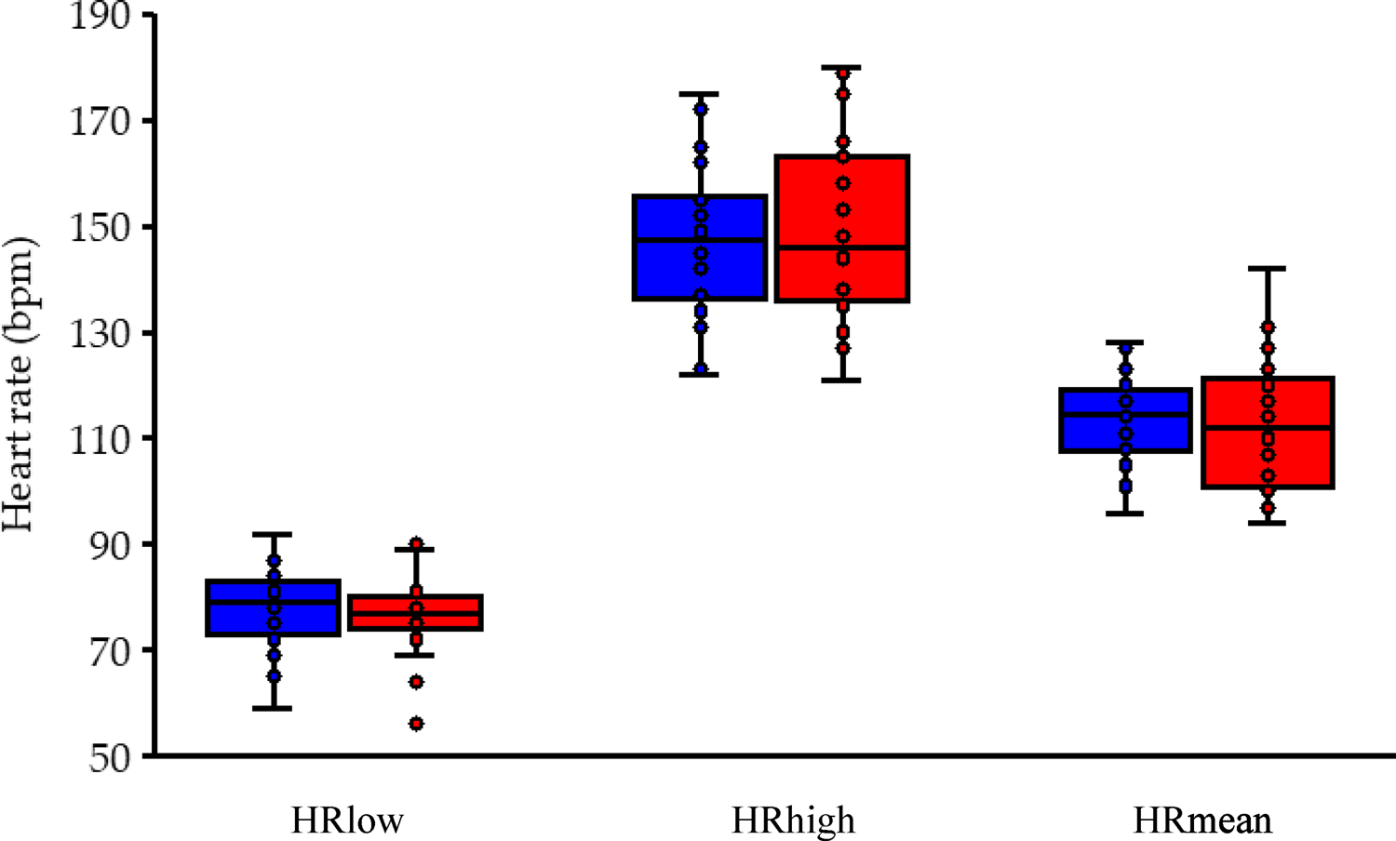
Box plots of HRlow, HRhigh, and HRmean during the swimming (blue boxes) and land-based (red boxes) activities. Each box represents the interquartile range, and the center line represents the median. Whiskers extend to the most extreme data point that is no more than 1.5 times the interquartile range from the edge of the box (Tukey style). Dots represent individual values

Figure 1 presents the HRlow, HRhigh, and HRmean during the swimming and land-based activities. No significant differences were observed between the two activity types for any of these variables. Across both activities, the mean HRlow was 77 bpm, the mean HRhigh was 149 bpm, and the mean HRmean was 114 bpm.

**Fig. 2:**
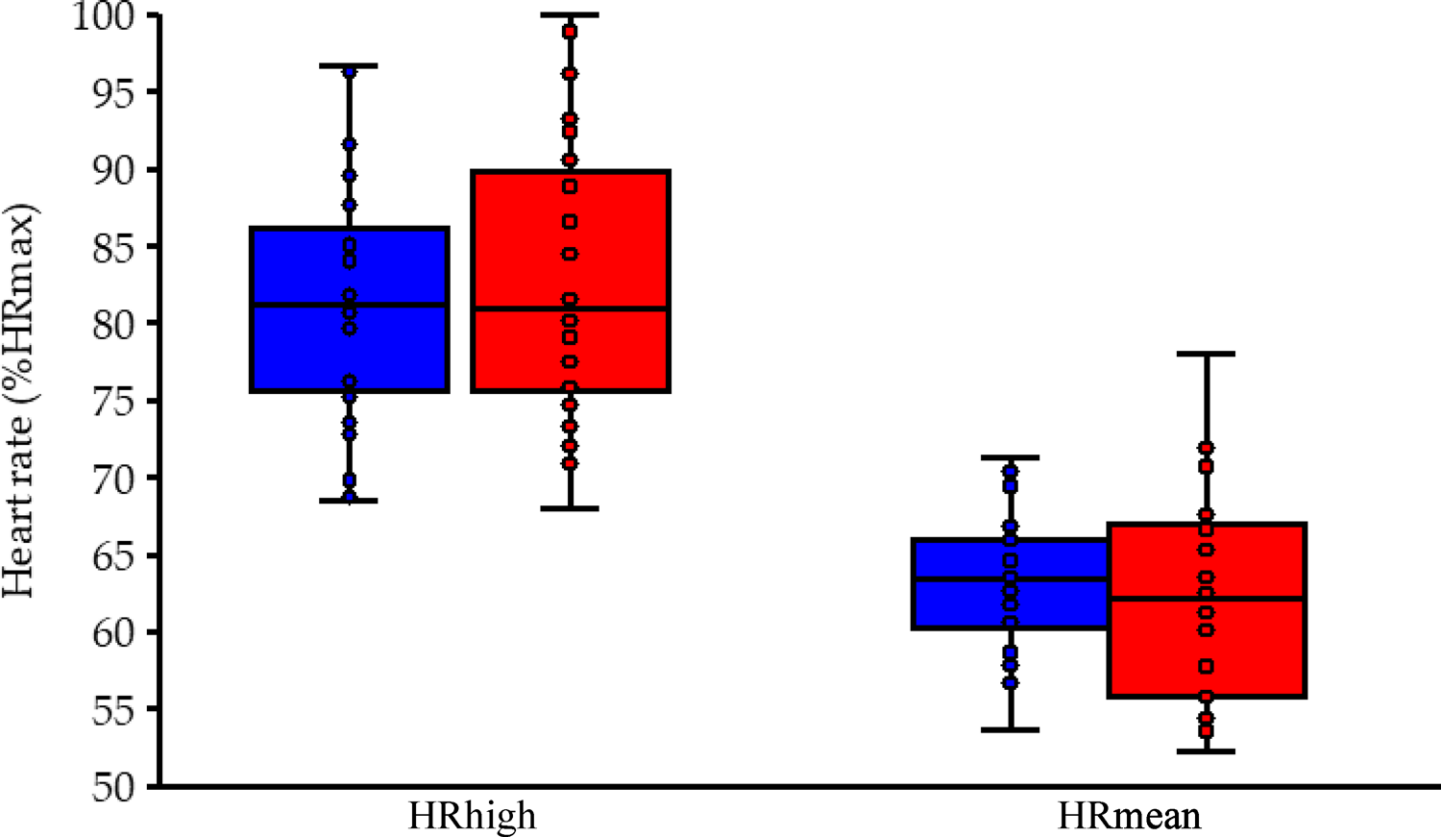
Box plots of HRhigh and HRmean, expressed as percentages of HRmax, during the swimming (blue boxes) and land-based (red boxes) activities. Each box represents the interquartile range, and the center line represents the median. Whiskers extend to the most extreme data point that is no more than 1.5 times the interquartile range from the edge of the box (Tukey style). Dots represent individual values.

Figure 2 presents the HRhigh and HRmean as percentages of HRmax. No significant differences were found between the two activity types for either variable.

**Fig. 3:**
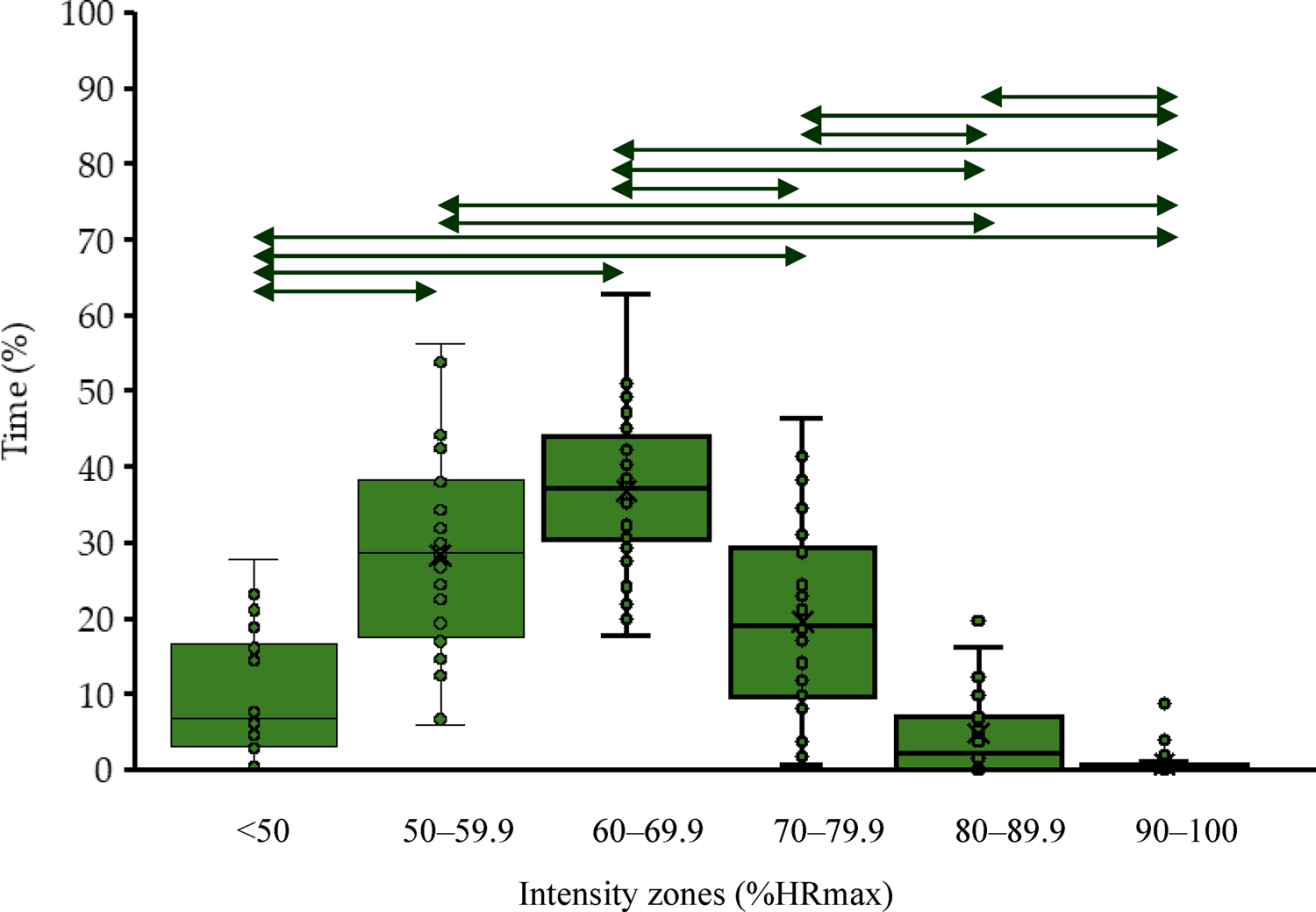
Box plots of time expended in each of the six intensity zones as a percentage of the total time of the swimming and land-based activities combined. Horizontal lines indicate statistically significant differences (*p* < 0.05). Each box represents the interquartile range, and the center line represents the median. Whiskers extend to the most extreme data point that is no more than 1.5 times the interquartile range from the edge of the box (Tukey style). Dots represent individual values

There was no significant interaction between activity type and intensity zone with regard to time expended in each zone (F₅,₂₅ = 1.294, *p* = 0.298, η² = 0.206), nor was there a significant main effect of activity (F₁,₂₉ = 1.673, *p* = 0.206, η² = 0.055). However, a significant main effect of intensity zone was observed (F₅,₂₅ = 475.138, *p* < 0.001, η² = 0.990). Given the absence of a main effect of activity, the combined data of the two activities are presented in Fig. 3.

Simple main effect analysis showed that participants expended significantly more time in the 60–69.9% HRmax zone (mean = 36.9%), compared with the < 50% (9.7%, *p* < 0.001), 70–79.9% (19.6%, *p* = 0.001), 80–89.9% (4.8%, *p* < 0.001), and 90–100% (0.7%, *p* < 0.001) zones. Time expended in the < 50% HRmax zone was significantly lower than in the 50–59.9% (*p* < 0.001) and 70–79.9% (*p* = 0.025) zones, and significantly higher than in the 90–100% zone (*p* < 0.001). Additionally, participants expended significantly more time in the 50–59.9% HRmax zone (28.4%), compared with the 80–89.9% and 90–100% zones (*p* < 0.001 for both), and significantly more time in the 70–79.9% HRmax zone, compared with the 80–89.9% and 90–100% zones (*p* < 0.001 for both). Finally, time expended in the 80–89.9% HRmax zone was significantly more than in the 90–100% zone (*p* < 0.001).

**Fig. 4:**
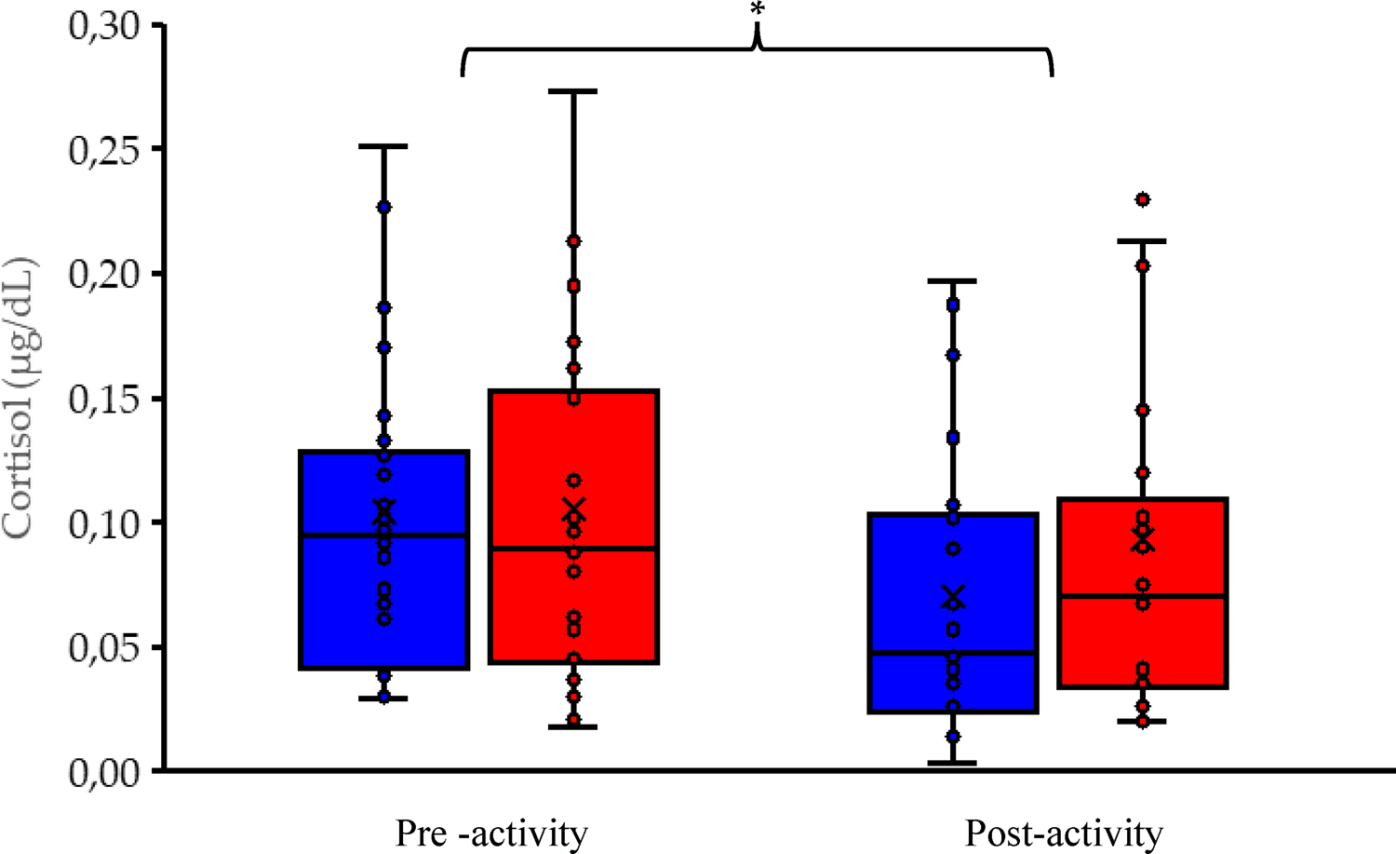
Salivary cortisol concentration before and after swimming (blue boxes) and land-based (red boxes) activities. Each box represents the interquartile range, and the center line represents the median. Whiskers extend to the most extreme data point that is no more than 1.5 times the interquartile range from the edge of the box (Tukey style). Dots represent individual values. *p = 0.012, significant main effect of time

There was no significant interaction between activity and time in the salivary cortisol concentration (F₁,₂₉ = 2.185, *p* = 0.150, η² = 0.070), nor was there a significant main effect of activity (F₁,₂₉ = 0.516, *p* = 0.478, η² = 0.017). However, a significant main effect of time was found (F₁,₂₉ = 7.202, *p* = 0.012, η² = 0.199), with cortisol decreasing after the activities (from 0.105 ± 0.076 to 0.082 ± 0.076 µg/dL, Fig. 4).

**Fig. 5:**
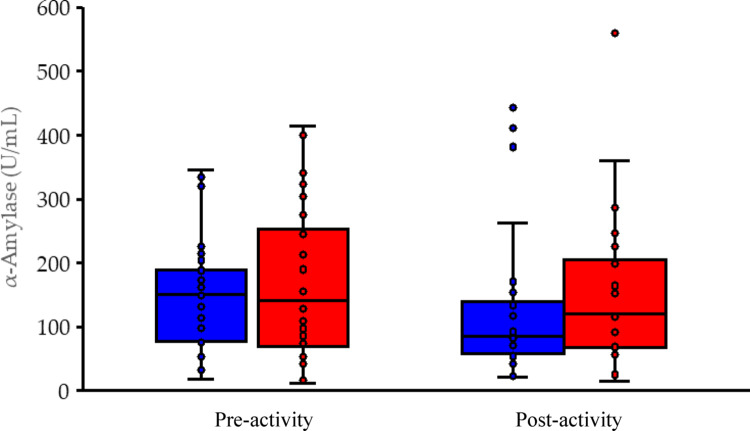
Salivary α-amylase concentration before and after swimming (blue boxes) and land-based (red boxes) activities. Each box represents the interquartile range, and the center line represents the median. Whiskers extend to the most extreme data point that is no more than 1.5 times the interquartile range from the edge of the box (Tukey style). Dots represent individual values

Similarly, the salivary α-amylase concentration showed no significant interaction between activity and time (F₁,₂₉ = 0.069, *p* = 0.795, η² = 0.002) or main effect of activity (F₁,₂₉ = 1.174, *p* = 0.288, η² = 0.039). A non-significant decrease in α-amylase was observed, from 157 ± 101 U/mL before the activities to 137 ± 112 U/mL (F₁,₂₉ = 2.999, *p* = 0.094, η² = 0.094) after their completion (Fig. 5).

**Fig. 6:**
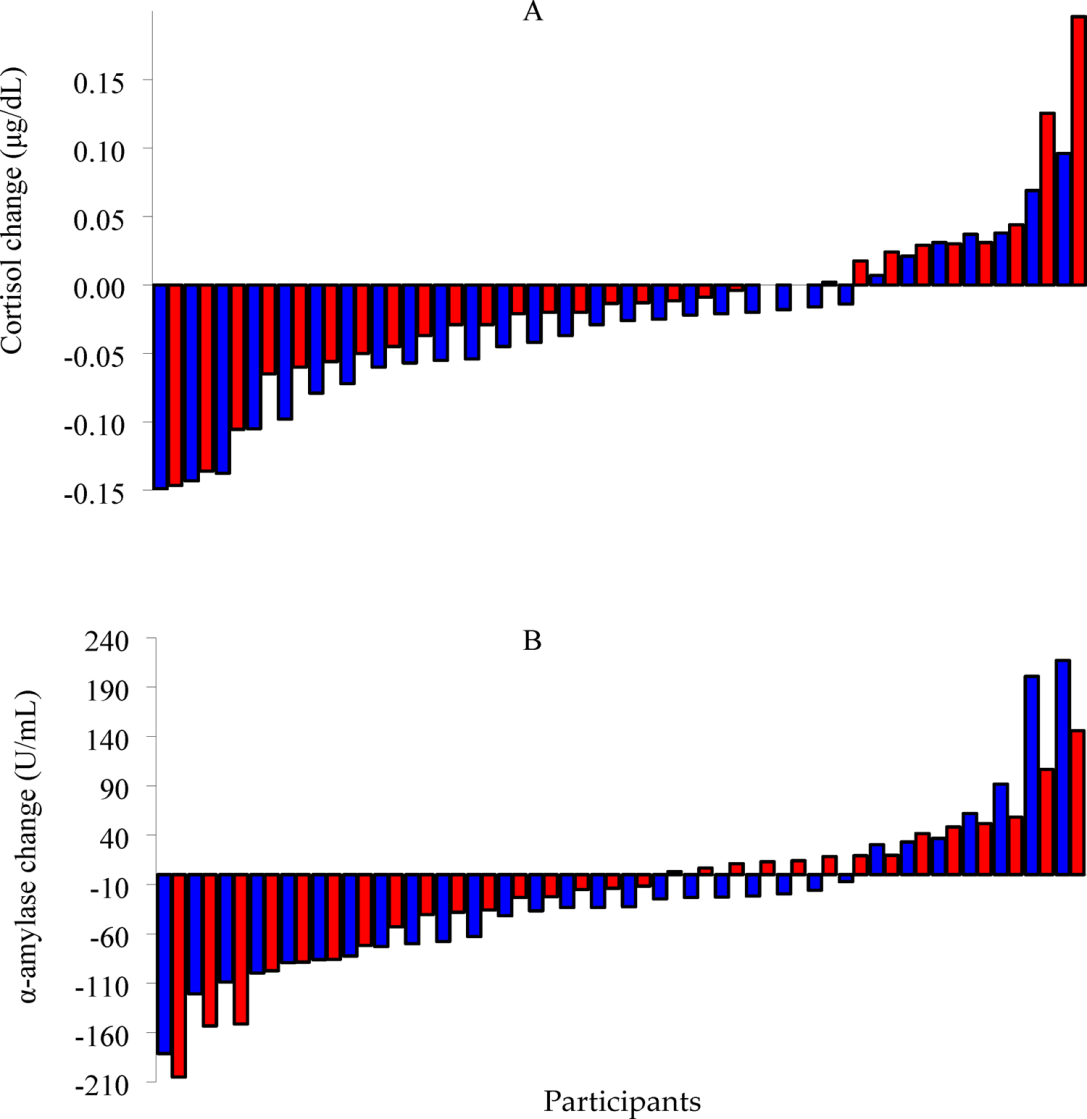
Individual variability in salivary cortisol (**A**) and α-amylase (**B**) concentration change following swimming (blue bars) and land-based activities (red bars). The changes are presented in ascending order for each activity; adjacent values do not correspond to the same participant

A remarkable interindividual variability was observed in the salivary cortisol and α-amylase responses to activity. Consistent with the statistical results mentioned above, most participants exhibited lower concentrations in both variables following each activity (Fig. 6).

**Fig. 7:**
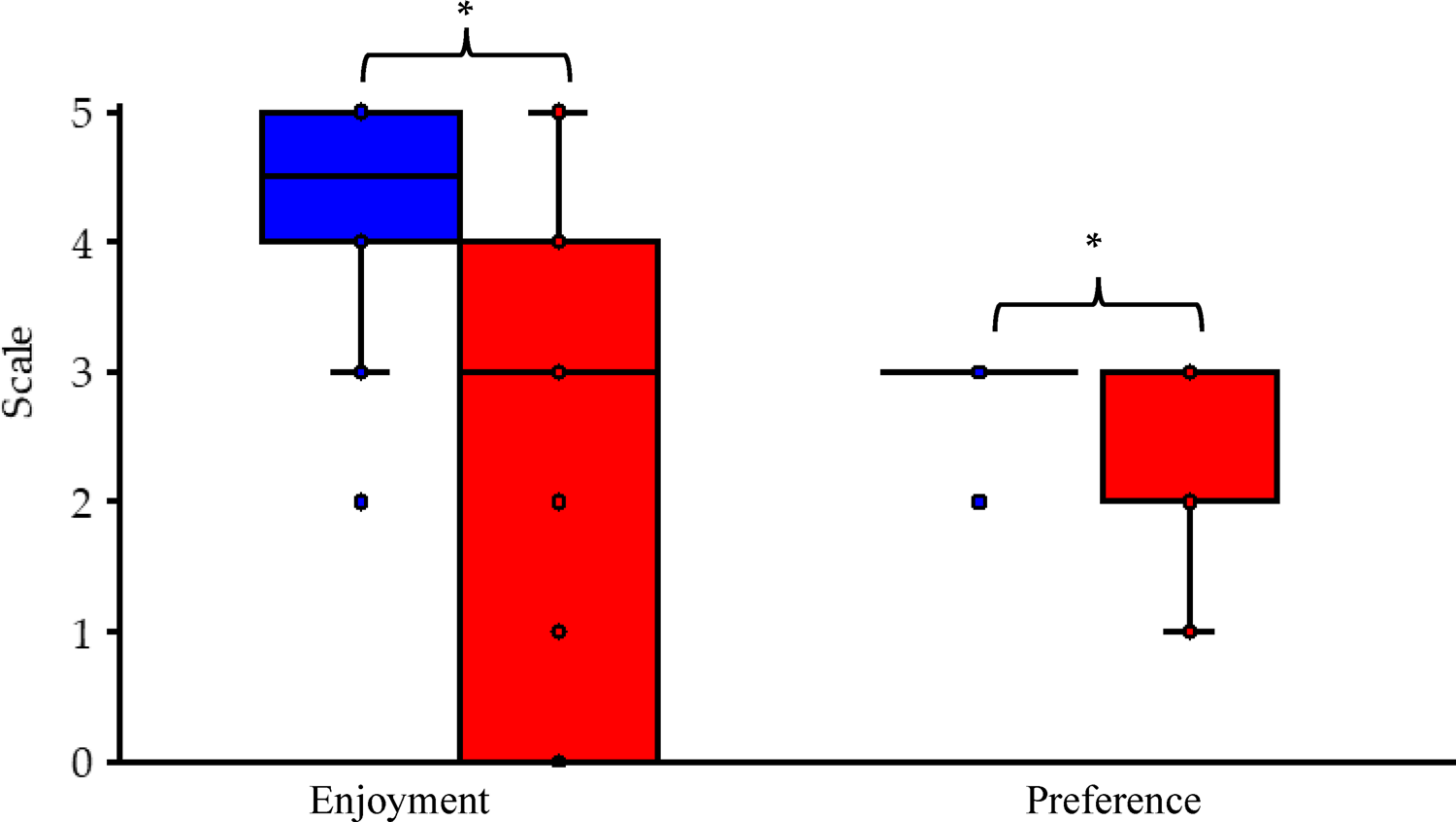
Levels of enjoyment on the CAPE scale and preference on the PAC scale, as reported by the participants for the swimming (blue box) and land-based (red box) activities. Each box represents the interquartile range, and the center line (top line in the last box) represents the median. Whiskers extend to the most extreme data point that is no more than 1.5 times the interquartile range from the edge of the box (Tukey style). Dots represent individual values. *p < 0.001

Participants reported significantly greater enjoyment of swimming compared to land-based activities (4.2 ± 1.0 vs. 2.5 ± 1.9, *p* < 0.001) and indicated a stronger preference for swimming (2.9 ± 0.4 vs. 2.4 ± 0.7, *p* < 0.001; Fig. 7).

Table 2 presents the most meaningful of the significant correlations identified. Negative correlations were found between biological age and HRmean during swimming (1), swimming time in the 70–79.9% HRmax zone (2), and HRhigh during land-based activity (3). Positive correlation was found for time in the 80–89.9% HRmax zone during the land-based activities with the post- activity cortisol concentration (4). A negative correlation was observed between preference for swimming and pre-swimming cortisol concentration, while a positive correlation was found with the change in cortisol concentrations during swimming (5). Finally, positive correlations were noted between enjoyment of swimming and enjoyment of land-based activities (6), as well as between preference and enjoyment of swimming (7).

**Table 2:** Significant correlations identified in the study

			Pre		Post		Change	
			*r*	*p*	*r*	*p*	*r*	*p*
1	Swimming	BA – HRmean	–0.415	0.023				
2	Swimming	BA – 70–79.9.9% HRmax	–0.418	0.022				
3	Land-based	BA – HRhigh	–0.367	0.046				
4	Land-based	Cortisol – 80–89.9.9% HRmax			0.558	0.001		
5	Swimming	Cortisol – PS	–0.437	0.016			0.382	0.037
6	Enjoyment	Swimming – land-based	0.404	0.027				
7	Swimming	Enjoyment – preference	0.712	< 0.001				

*BA:* biological age; *HRmean:* mean heart rate; *HRhigh:* highest heart rate; *PS:* preference for swimming; *x–y% HRmax:* percentage of activity time expended in the x-to-y% maximum heart rate zone

## Discussion

The aims of the present study were (i) to investigate the physiological and psychological responses of children and adolescents with ID and/or ASD during aquatic and land-based activity protocols and (ii) the relationship between physical activity type, stress biomarkers, and perceived enjoyment using HR responses, salivary cortisol and α-amylase measurements, and questionnaire-based assessment of enjoyment and preference. Our main findings were that (i) HR responses did not differ significantly between activities, (ii) salivary cortisol concentrations decreased after both activities, with no difference between them, and (iii) participants reported greater enjoyment of, and a stronger preference for swimming than land-based activities.

Compared with previous studies, the similarity of cardiac responses between swimming and land-based activities in the present study suggests comparable exercise intensity levels. This aligns with findings in healthy adults showing similar cardiac output during aquatic and land-based activities (Hauber et al. 1997), although some studies have reported reduced cardiac strain during swimming (Benelli et al. 2004). These discrepancies may be attributed to differences in exercise protocols (e.g., duration, intensity regulation, environmental conditions) and participant characteristics, including age, diagnosis, fitness level, and degree of intellectual disability. Taken together, these findings suggest that cardiac responses to exercise in individuals with ID or ASD are strongly influenced by population-specific factors, limiting direct comparisons with studies conducted in typically developing individuals. Research involving individuals with ID or ASD remains limited, underscoring the originality of the present study.

The average HRhigh of 149 bpm during the two activities cannot be directly compared with existing literature, which primarily focuses on different populations. In individuals with DS, cardiac response tends to remain below typical values, even after strenuous activity (Merati et al. 2024). Conversely, higher HR values have been observed in children with ASD during swimming (Lawson et al. 2020), likely influenced by age, while lower HR values are reported during land-based activities (Pace and Bricout 2015). A consistent 9 bpm deficit in peak HR in individuals with ID, compared with age-matched peers, has been documented (Hilgenkamp and Baynard 2018). Reduced cardiac responses may result from a combination of low motivation, limited comprehension, physical inactivity, and autonomic dysfunction (Fernhall and Pitetti 2001). Differences between the present findings and previous studies are likely attributable to variations in sample composition (age range, diagnosis, functional level), exercise program design (continuous vs. intermittent activity, familiarity with the task), and environmental context (aquatic vs. land-based), all of which are known to influence cardiovascular responses in special populations.

The average HRmean of 114 bpm during the two activities—equivalent to 63% of the theoretical HRmax—indicates moderate intensity. This agrees with values reported during swimming in children with ASD (Lawson et al. 2020) and falls within recommended exercise intensity ranges (60–80% HRmax) for 20–60 min (Casey et al. 2010; Wu et al. 2017; Jacinto et al. 2023). According to current exercise guidelines for special populations, moderate-intensity aerobic activity is defined as 60–80% of HRmax; therefore, the intensity achieved in the present study fully complies with established standards, supporting the appropriateness and safety of the applied exercise protocols. The minimal time expended in the 90–100% HRmax zone (0.7%) is consistent with well-documented limitations in sustaining high-intensity exercise among individuals with ID, often linked to lower cardiorespiratory and muscular capacity (Boonman et al. 2019).

In this study, both swimming and land-based activities resulted in comparable, large (in terms of effect size) reductions in salivary cortisol, indicating decreased stress levels. These findings are consistent with previous studies reporting cortisol reductions following exercise interventions in both aquatic and terrestrial environments (Lundqvist et al. 2022; Durán-Carabali et al. 2021; Abe et al. 2002). Given the pronounced diurnal variation of cortisol, characterized by higher concentrations in the morning and lower concentrations in the evening, each participant completed both activities at the same time of day. The observed decline in the salivary cortisol concentrations following both activities may therefore be attributed either to the normal diurnal decrease over time or to the moderate exercise intensity, as indicated by participants’ heart rate responses. Moderate-intensity exercise, particularly when perceived as non-stressful or familiar, has been shown to reduce HPA axis activity and sympathetic nervous system activation (Hackney 2006). In contrast to cortisol, salivary α-amylase concentrations typically increase over the course of the day (Nater et al. 2007). Therefore, the observed decrease in salivary α-amylase concentrations following both activities is likely attributable to the relaxing effects of the exercises on the participants (Ali and Nater 2020). By measuring both cortisol and salivary α-amylase in the same saliva samples, we were able to simultaneously assess the activity of the HPA axis and the autonomic nervous system. Results from both biomarkers indicate that the activities reduced stress in children and adolescents with ID and/or ASD.

The absence of any statistically significant correlation between cortisol and α-amylase at any measurement point or between the changes in the two variables is consistent with previous findings (Allwood et al. 2011; Schumacher et al. 2013), indicating that these biomarkers do not usually change in parallel. Evidence of concurrent changes in individuals with neurodevelopmental disorders remains limited (Anesiadou et al. 2021; Durán-Carabali et al. 2021; Keller et al. 2012). For instance, children diagnosed with neurodevelopmental disorders exhibited cortisol secretion patterns analogous to those observed in typically developing peers but different diurnal patterns of α-amylase (Anesiadou et al. 2021). Moreover, cortisol and α-amylase had opposite diurnal patterns (Nater and Rohleder 2009), while a physical therapy session using the neurodevelopmental technique in children with cerebral palsy revealed no significant pre-post changes in either biomarker (Durán-Carabali et al. 2021). Collectively, these findings support the notion that the HPA axis and autonomic nervous system operate through partially independent mechanisms, and that stress responses in neurodevelopmental populations cannot be adequately explained by linear or uniform response models (Keller et al. 2012). Given the lack of a significant correlation between cortisol and α-amylase in the present study, further research in individuals with neurodevelopmental disorders is needed to better understand the independent mechanisms and interactions of the HPA axis and autonomic nervous system. Although current evidence remains limited, additional studies focusing on neurodevelopmental disorder populations will help clarify the independent regulation and potential interactions of these two stress systems.

The present study’s findings from the CAPE and PAC tools highlight swimming as a preferred and more enjoyable activity for children and adolescents with ID and ASD, compared with land-based activity. This preference probably reflects the unique physical and psychosocial affordances of the aquatic environment. According to prior work (Eversole et al. 2016), children with ASD report higher enjoyment levels in swimming than most physical activities, rivaling their engagement with popular recreational pursuits. Enhanced enjoyment likely derives from the calming, sensory-regulating properties of water, which create a safe and supportive context for motor engagement—factors that are especially critical for this population.

Beyond enjoyment, swimming has been shown to yield broad functional benefits, improving motor competence and self-confidence in populations with neurodevelopmental challenges, such as cerebral palsy (Declerck et al. 2016). Moreover, swimming is recognized as an effective and enjoyable activity that improves cardiorespiratory health and supports lifelong physical activity engagement across populations with and without disabilities (Fragala-Pinkham et al. 2008, 2011).

At the physiological and biochemical levels, the negative correlation of biological age with HRmean during swimming, time expended in the 70–79.9% HRmax zone, and HRhigh during land-based activities agrees with a study in female athletes, which found stronger cardiovascular responses to moderate-to-high intensity exercise in younger individuals (Nikolaidis et al. 2015). The positive correlation of cortisol after the land-based activity with time expended in the 80–89.9% HRmax zone reinforces the intensity-dependent activation of the HPA axis during exercise (Anderson et al. 2016; Hill et al. 2008). The negative correlation between swimming preference and pre-swimming cortisol, coupled with the positive association between swimming preference and cortisol change after swimming, suggests that emotional anticipation may modulate stress responses (Borges et al. 2021; Gerber and Colledge 2023; van Paridon et al. 2017).

Enjoyment of swimming and land-based activities was positively correlated, suggesting that the attitude toward physical activity among participants was general, not activity-specific. This finding is in agreement with theoretical frameworks emphasizing intrinsic motivation as a key driver for enhancing exercise experiences and sustaining long-term participation (Carron et al. 2005; Lonsdale et al. 2008). The observed association between preference for swimming and enjoyment from both activity types suggests that satisfaction derived from one form of movement may extend to others, particularly when a positive disposition toward physical activity exists (Deci and Ryan 2000).

In conclusion, the present study showed that swimming and land-based activities constituted moderate-intensity exercise, as evidenced by cardiac responses, and reduced physiological and/or mental stress, reflected in decreased post-activity salivary cortisol, in children and adolescents with ID and ASD. Land-based activities are more popular, and aquatic activities are suitable for individuals with high stress levels. The potential reasons for the greater preference for swimming may include the calming environment and the improvement of self-efficacy, which need to be further verified by subsequent research. These findings underscore the importance of enjoyment and tailored interventions to support sustained engagement and improve psychosocial outcomes in children and adolescents with intellectual disabilities.

### Future directions

Future studies should involve larger and more diverse samples to improve generalizability and to examine the effects of age, sex, and type of disability on exercise responses. Exploring a wider range of exercise modalities, intensities, and durations is necessary to identify optimal and individualized intervention profiles. The use of wearable and non-invasive monitoring technologies, combined with qualitative approaches, may further enhance the understanding of both physiological and subjective responses to exercise.

## Data Availability

The data that support the findings of this study are available from the corresponding author upon request.
